# Sacubitril/Valsartan Reduces Fibrosis and Alleviates High-Salt Diet-Induced HFpEF in Rats

**DOI:** 10.3389/fphar.2020.600953

**Published:** 2021-01-14

**Authors:** Wenchao Zhang, Jianwei Liu, Yang Fu, Huifang Ji, Zheyan Fang, Wanming Zhou, Huimin Fan, Yingxuan Zhang, Yan Liao, Ting Yang, Xiaolin Wang, Wanwan Yuan, Xiaoshu Chen, Yi-fei Dong

**Affiliations:** ^1^ Department of Cardiovascular Medicine, the Second Affiliated Hospital of Nanchang University, Nanchang, China; ^2^ Chang Xing People’s Hospital, Huzhou, China

**Keywords:** heart failure with preserved ejection fraction, vascular injury, sacubitril/valsartan, fibrosis, high-salt diet

## Abstract

Previous studies have confirmed the clinical efficacy of sacubitril/valsartan (Sac/Val) for the treatment of heart failure with reduced ejection fraction (HFrEF). However, the role of Sac/Val in heart failure with preserved ejection fraction (HFpEF) remains unclear. Sac/Val is a combination therapeutic medicine comprising sacubitril and valsartan that acts as a first angiotensin receptor blocker and neprilysin inhibitor (angiotensin-receptor neprilysin inhibitor (ARNI)). Here, we investigated the role of Sac/Val in high-salt diet-induced HFpEF coupled with vascular injury as well as the underlying mechanism. Rats were fed with high-salt feed, followed by intragastric administration of Sac/Val (68 mg/kg; i.g.). The results of functional tests revealed that a high-salt diet caused pathological injuries in the heart and vascular endothelium, which were significantly reversed by treatment with Sac/Val. Moreover, Sac/Val significantly decreased the levels of fibrotic factors, including type I collagen and type Ⅲ collagen, thus, reducing the ratio of MMP2/TIMP2 while increasing Smad7 levels. Further investigation suggested that Sac/Val probably reversed the effects of high-salt diet-induced HFpEF by inhibiting the activation of the TGF-β1/Smad3 signaling pathway. Thus, treatment with Sac/Val effectively alleviated the symptoms of high-salt diet-induced HFpEF, probably by inhibiting fibrosis via the TGF-β1/Smad3 signaling pathway, supporting the therapeutic potential of Sac/Val for the treatment of HFpEF.

## Introduction

Heart failure (HF) with preserved ejection fraction (HFpEF) is a peculiar clinical phenotype of HF, which involves the typical signs/symptoms of HF coupled with an EF of >50% (Hogg et al., 2004). The pathophysiological changes related to this disease include increased left ventricular (LV) filling pressures, increased vascular injury, and weeny defects of systolic function despite relatively preserved EF (Tan et al., 2009; Borlaug and Paulus, 2011; Franssen et al., 2016). The development of HFpEF is mainly attributed to reactive fibrosis in the myocardium (Mohammed et al., 2015). Approximately half of the patients with HF have HFpEF that is associated with substantial morbidity and mortality, thus resulting in poor outcomes similar to those with HF with reduced ejection fraction (HFrEF) (Tsuchihashi-Makaya et al., 2009). Several existing treatments for HFrEF have shown promising outcomes; however, there are no effective therapies for HFpEF (Aurigemma and Gaasch, 2004; Bolam et al., 2018). Thus, it is necessary to find potential therapeutic targets that selectively inhibit reactive fibrosis for treating HFpEF.

Sacubitril/valsartan (Sac/Val), also known as LCZ696, is the first type of angiotensin-receptor neprilysin inhibitor (ARNI) used for the treatment of HFrEF (Almuflfleh et al., 2017). The global PARADIGM-HF (Prospective Comparison of ARNI With Angiotensin-Converting Enzyme Inhibitor to Determine Impact on Global Mortality and Morbidity in Heart Failure) study compared Sac/Val with enalapril in patients with HFrEF and found significant clinical benefits of Sac/Val for the treatment of HFrEF (McMurray et al., 2013). Sac/Val decreased the rate of mortality or hospitalization in HF by approximately 20% as well as increased survival by approximately 2 years in HFrEF patients (McMurray et al., 2014; Claggett et al., 2015). Therefore, the American College of Cardiology/American Heart Association/Heart Failure Society of America renewed their medical guidelines to recommend (Class I) the use of Sac/Val to reduce the associated morbidity and mortality in patients with HFrEF (Kusaka et al., 2015). A study found that Sac/Val exerted its functions mainly by decreasing the pathological fibrosis and myocardial hypertrophy (Vaskova et al., 2020). One clinical study reported that Sac/Val did not significantly lower the total rate of hospitalizations and death due to HF in patients with HFpEF (Solomon et al., 2019). On the contrary, the PARALLAX trial recently concluded that Sac/Val showed some improvement in patients with HFpEF (Shah et al., 2019).

Previous studies have shown that HFpEF and HFrEF share some pathophysiological characteristics, such as fibrosis (Hahn et al., 2020). Thus, Sac/Val could serve as a novel therapeutic agent for the treatment of HFpEF as well. Here, we investigated the therapeutic potential and the underlying mechanism of Sac/Val in the treatment of HFpEF coupled with vascular injury.

## Materials and Methods

### Materials

Male Dahl/salt-sensitive (Dahl/SS) and SS-13BN rats (6 weeks old, 200–250 g) were procured from Beijing Charles River Laboratory Animal Co., Ltd. (Beijing, China). Powdered Sac/Val and valsartan were purchased from Novartis Pharma AG Co., Ltd. (Beijing, China). Low-salt feed (0.3% NaCl) and high-salt feed (8% NaCl) were acquired from Beijing Keao Xieli Feed Co., Ltd. (Beijing, China). The following antibodies were used: anti-Collagen type I (Cat# 14695-1-AP), anti-Collagen type Ⅲ (Cat# 22734-1-AP), anti-TGF-β1 (Cat# 21898-1-AP), anti-Smad3 (Cat# CST-5678S), anti-Smad7 (Cat# 25840-1-AP), anti-MMP2 (Cat# ab97779), anti-TIMP2 (Cat# ab180630), and anti-GAPDH (Cat# 60004-1-Ig). The enzyme-linked immunosorbent assay (ELISA) kits were purchased from MultiSciences Biotech Co., Ltd. (Wuhan, China) and were used to detect the levels of BNP, NT-ProBNP, and NO in serum samples of rats.

### Animal Model

This study included 40 male rats (30 Dahl/SS rats and 10 SS-13BN rats) who were housed at 22 ± 3°C at a relative humidity of 55 ± 5%, exposed to a 12-h light/dark cycle, and had free access to water and food. The rats were fed low-salt feed (0.3% NaCl) for one week to adapt to the housing conditions before study initiation. All experimental treatments complied with the guidelines of the Animal Care and Use Committee of the Second Affiliated Hospital of Nanchang University, China.

After the adaptation period, the Dahl/SS rats were fed with high-salt feed (8% NaCl) until they were 19 weeks old, thus constructing an animal model of HFpEF. Then, these 30 male Dahl/SS rats were randomly divided into three groups: 1) high-salt group (Saline, n = 10): this group was continuously fed with high-salt feed (8% NaCl) and received no further treatment; 2) Sac/Val group (n = 10): this group received high-salt feed (8% NaCl) and Sac/Val at 8 a.m. everyday intragastrically (68 mg/kg, i.g.); 3) valsartan group (Val, n = 10): this group received high-salt feed (8% NaCl) and Val at 8 a.m. everyday intragastrically (31 mg/kg, i.g.). On the contrary, the SS-13BN rats were fed with low-salt feed (0.3% NaCl) throughout the experimental period until they were sacrificed and were classified as the control group (Control, n = 10) (Wang et al., 2016). Body weight (BW), blood pressure, echocardiography, and other parameters were recorded at different time points. We also observed the living conditions of the rats and comprehensively judged whether the animal model of HFpEF was successfully constructed.

### Echocardiography and Detection of Blood Pressure

Echocardiography was performed to evaluate the cardiac function of all rats using a Vevo770 (VisualSonics, Toronto, Canada) equipped with a 30 Hz transducer. First, the rats were anesthetized by injecting 2% isoflurane. Next, they were fixed on a heating mat in the supine position, and their chest hair was shaved using a razor. Then, we used the short-axis view following M-mode ultrasound to detect left ventricle (LV) internal dimensions at the end of diastole (LVIDd). We also detected interventricular septum thickness at the end of diastole (IVSd), left ventricular posterior wall thickness at the end of diastole (LVPWd), left atrial internal dimensions (LA), left ventricular ejection fraction (LVEF), and left ventricular fractional shortening (LVFS). Furthermore, we switched the device to the color Doppler ultrasound mode to measure maximum peak blood flow velocity at the early phase of diastole (E) and maximum peak blood flow velocity at the end of diastole (A) in the mitral orifice via the four-chamber view. Finally, we detected the E′ peak and the A′ peak in the mitral orifice using tissue Doppler ultrasound. All measurements were recorded at 7, 13, 19, and 23 weeks, and the final value was an average of six repeated measurements.

After fixing the rats, a BP-2010E noninvasive rat tail sphygmomanometer (Softron, Japan) was used to detect their blood pressure by attaching the transducer to 3 cm of rats’ tail root. The operating principle of the device involved the detection of the pulse in the tail artery of the rat to observe the corresponding waveform. We recorded both systolic and diastolic blood pressures, and the device automatically calculated the average arterial pressures. The final values were derived from the mean of six repeated measurements.

### Measurement of Hemodynamics

A multichannel biological recorder (Chengdu Instrument Company, Sichuan, China) was connected with the arterial cannula and blood pressure transducer for carotid artery intubation. The process of carotid artery intubation was as follows: First, the rats were anesthetized by injecting 20% urethane intraperitoneally and fixed on a heating pad that could maintain the body temperature of the rats. Then, the right carotid artery was isolated, the distal end was ligated, and the proximal end was clipped. A V-shaped incision was made in the middle of carotid artery occlusion. After the arterial cannula was inserted into the right carotid artery, the clamp was released and the arterial cannula was reversed. We observed and collected the waveform in the device during the insertion of the arterial cannula. When the arterial cannula was inserted into the left ventricle and the lowest point of the waveform dropped to 0 mmHg, the procedure was terminated. Finally, the datum was used to compute hemodynamics, such as left ventricular end-diastolic pressure (LVEDP), left ventricular relaxation time constant (Tau), and maximum left ventricular pressure decreasing rate (−dp/dt_max_). After the operation, rats were immediately sacrificed and the tissues were collected for further experiments.

### Measurement of Endothelium-Dependent Vasorelaxation

We used DMT620 (Softron, Japan) and the Krebs–Henseleit solution to detect endothelium-dependent vasorelaxation. The carotid artery, isolated from rats, was strung on the wire of DMT620, filled with Krebs–Henseleit solution, followed by data collection. Next, we used acetylcholine (Ach) and sodium nitroprusside (SNP) to measure endothelium-dependent and endothelium-independent vasodilation of the carotid artery, respectively.

### ELISA

The serum levels of BNP, NT-ProBNP, and NO were measured using BNP (Cat# CSB-E07972r, Wuhan, China), NT-ProBNP (Cat# ER0309, Wuhan, China), and NO ELISA kits (Cat# A013-2-1, Nanjing, China) following the manufacturer’s guidelines.

### Histopathological Assessment of Heart and Vascular Endothelium Tissues

After excising the heart and the vascular endothelium tissues of the rats, they were washed thrice with ice-cold saline. The tissues were fixed in 4% formaldehyde solution for 1 h, followed by hematoxylin and eosin (H&E) staining, Masson’s trichrome (Masson) staining, and immunohistochemical (IHC) analyses. The remaining tissue specimens were stored at −80°C for further investigation of the molecular mechanisms.

### RNA Extraction and Quantitative Real-Time- (qRT-) PCR

TRIzol reagent (Cat# DP424, Tiangen Biotech, China) was used to extract total RNA from the heart tissue specimens, followed by quantification using NanoDrop 2000c Spectrophotometer (Thermo Fisher Scientific, Inc.). Then, the cDNA transcription was completed using the first-strand cDNA synthesis kit (Cat# KR116-02, Tiangen Biotech, China). Next, a PCR thermocycler (ABI 7900TH, United States) was used to perform qRT-PCR using SYBR green as the fluorescence dye (Cat# FP205-02, Tiangen Biotech, China) with a reaction volume of 20 μL following the manufacturer’s instructions. GAPDH was used as the internal reference. The data collected from the device were used to calculate the relative gene expression using the 2^−△△CT^ method. Table 1 lists the primers of the target genes used in this study, which were designed and synthesized by Shanghai Sangon Biotech Engineering Co., Ltd., Shanghai, China.

**TABLE 1:** Primer sequences used in qRT-PCR experiment in this study.

Gene	Primer	Sequence
Collagen 1	Forward primer	GAG​CGG​AGA​GTA​CTG​GAT​CGA
	Reverse primer	CTG​ACC​TGT​CTC​CAT​GTT​GCA
Collagen 3	Forward primer	TGC​CAT​TGC​TGG​AGT​TGG​A
	Reverse primer	GAA​GAC​ATG​ATC​TCC​TCA​GTG​TTG​A
TGF-β1	Forward primer	CAA​CAA​TTC​CTG​GCG​TTA​CCT
	Reverse primer	GCC​CTG​TAT​TCC​GTC​TCC​TT
Smad 7	Forward primer	GGC​TTT​CAG​ATT​CCC​AAC​TT
	Reverse primer	ACA​CCC​ACA​CAC​CAT​CCA​CG
GAPDH	Forward primer	CCT​CGT​CCC​GTA​GAC​AAA​ATG
	Reverse primer	TGA​GGT​CAA​TGA​AGG​GGT​CGT

### Western Blot Analysis

A protein extraction kit (Cat# P0013B, Beyotime Biotechnology, Jiangsu, China) was used to extract total proteins from the tissue specimens of the rats’ heart, followed by quantification using the BCA protein assay kit (Cat# P0012, Beyotime Biotechnology, Jiangsu, China). Next, 10% sodium dodecyl sulfate-polyacrylamide gel electrophoresis (SDS-PAGE) was used to separate the proteins, which were then transferred to PVDF membranes (Millipore, Bedford, United States). Then, the membranes were incubated at 4°C with specific primary antibodies for more than 12 h, followed by incubation with appropriate secondary antibodies, including goat anti-rabbit IgG or goat anti-mouse IgG (ZSGB-Bio, Peking, China, 1:5,000). The primary antibodies used in the present study were as follows: anti-Collagen type I (1:1,000), anti-Collagen type Ⅲ (1:1,000), anti-TGF-β1 (1:1,000), anti-Smad3 (1:1,000), anti-Smad7 (1:1,000), anti-MMP2 (1:1,000), anti-TIMP2 (1:1,000), and anti-GAPDH (1:1,000). Finally, we used the enhanced chemiluminescence (ECL) detection kit to detect protein bands using an ECL scanner (Thermo Fisher Scientific). Western blotting was repeated at least thrice, and the Image Lab 4.0.1 software was used for final analysis.

### Statistical Analysis

Statistical analysis was performed using GraphPad Prism 7.0 software (GraphPad Software Inc., CA, United States). Student’s *t*-test was used to compare two groups, while the one-way ANOVA was used to compare multiple groups. Data were presented as mean ± standard deviation (SD), and *p* value < 0.05 was considered statistically significant.

## Results

### High-Salt Diet Induced the Occurrence of HFpEF

**FIGURE 1:**
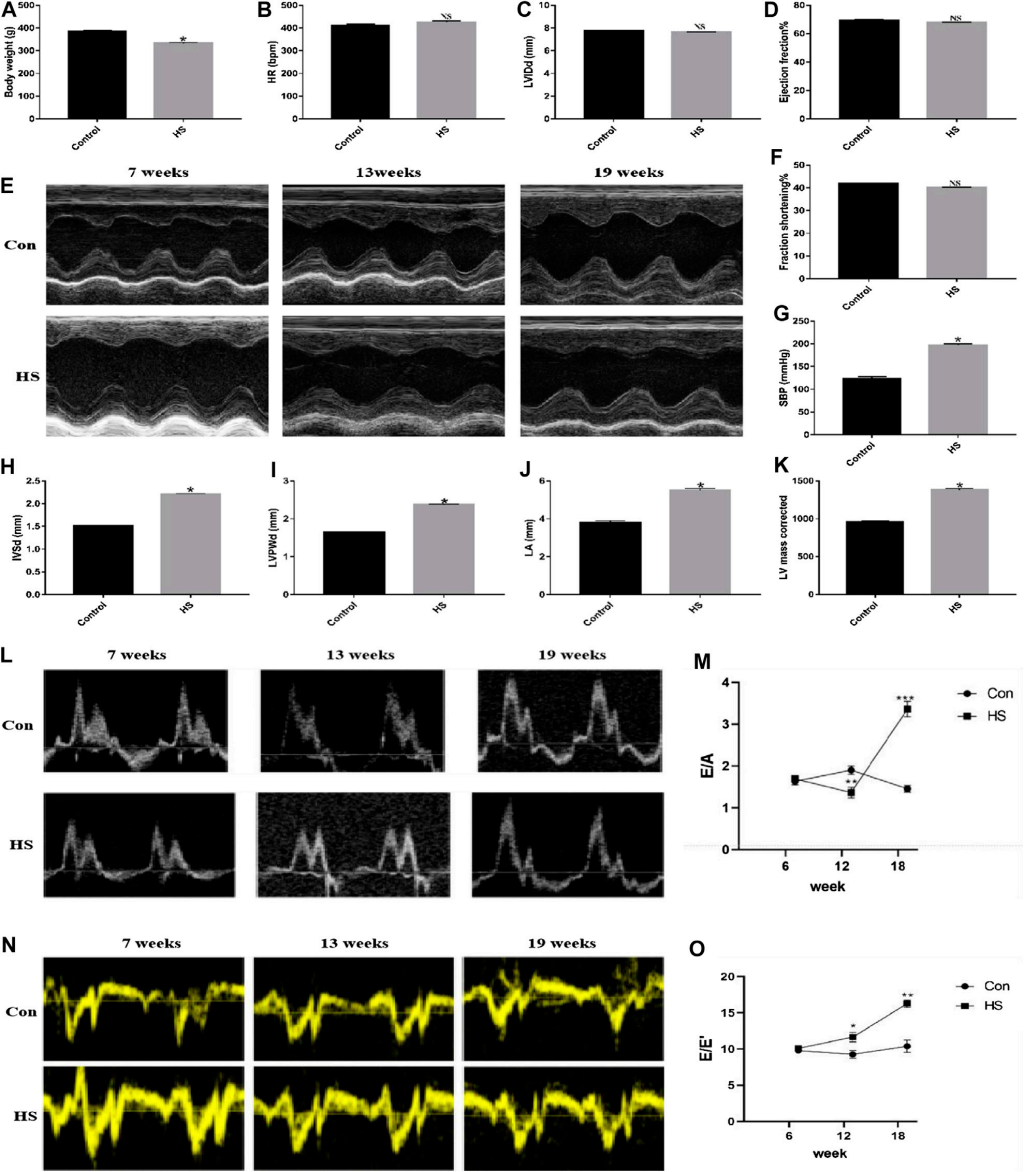
The animal model of HFpEF was successfully constructed after feeding the rats with high-salt feed (8% NaCl) until 19 weeks. **(A)** Body weight was measured to evaluate general condition of rats (n = 10 in each group). **(E)** Echocardiographic analysis was performed to assess the cardiac function of rats in different weeks. **(B–D, F–K)** Different parameters such as HR, LVIDd, EF%, FS%, SBP, IVSd, LVPWd, LA, and LV mass corrected were obtained via many measurements (echocardiography and detection of blood pressure). **(L–O)** The ratios of E/A and E/E′ were collected through color Doppler ultrasound. *p < 0.05 means statistic significance; NS means no statistic significance. All these data represent the mean ± SD of at least three times of the experiment. HR, heart rate; LVIDd, left ventricle internal dimensions at the end of diastole; EF, left ventricular ejection fraction; FS, left ventricular fractional shortening; SBP, systolic blood pressure; IVSd, interventricular septum thickness at the end of diastole; LVPWd, left ventricular posterior wall thickness at the end of diastole; LA, left atrial internal dimensions; E, maximum peak blood flow velocity at early phase of diastole; A, maximum peak blood flow velocity at the end of diastole.

After 19 weeks, the rats in the HS groups exhibited typical HF signs/symptoms, such as bradykinesia, dull hair that shed easily, shortness of breath, and occasional coughing of frothy pink sputum compared with the Control group. Additionally, there was a significant increase in systolic blood pressure (SBP), IVSd, LVPWd, LA, and corrected LV mass (*p* < 0.05) in the HS groups than the Control group. Moreover, there was a significant decrease in BW in the HS groups (*p* < 0.05) than in the Control group. However, there was an insignificant change in the levels of LVEF, LVFS, LVIDd, and heart rate (HR) (*p* > 0.05) in the HS groups compared with the Control group (Figures 1A–K). These parameters indicated the successful construction of the animal model of HFpEF. Next, we evaluated the ratios of E/A and E/E′ as well as the changes in blood flow in the mitral orifice using the color Doppler ultrasound in 7-, 13-, and 19-week-old rats and found elevated ratios of E/A and E/E′ (*p* < 0.05) in the HS groups than in the Control group (Figures 1L–O), demonstrating that the high-salt diet induced HFpEF in rats.

### Treatment With Sac/Val Attenuated Cardiac Dysfunction Associated With High-Salt Diet-Induced HFpEF

**FIGURE 2:**
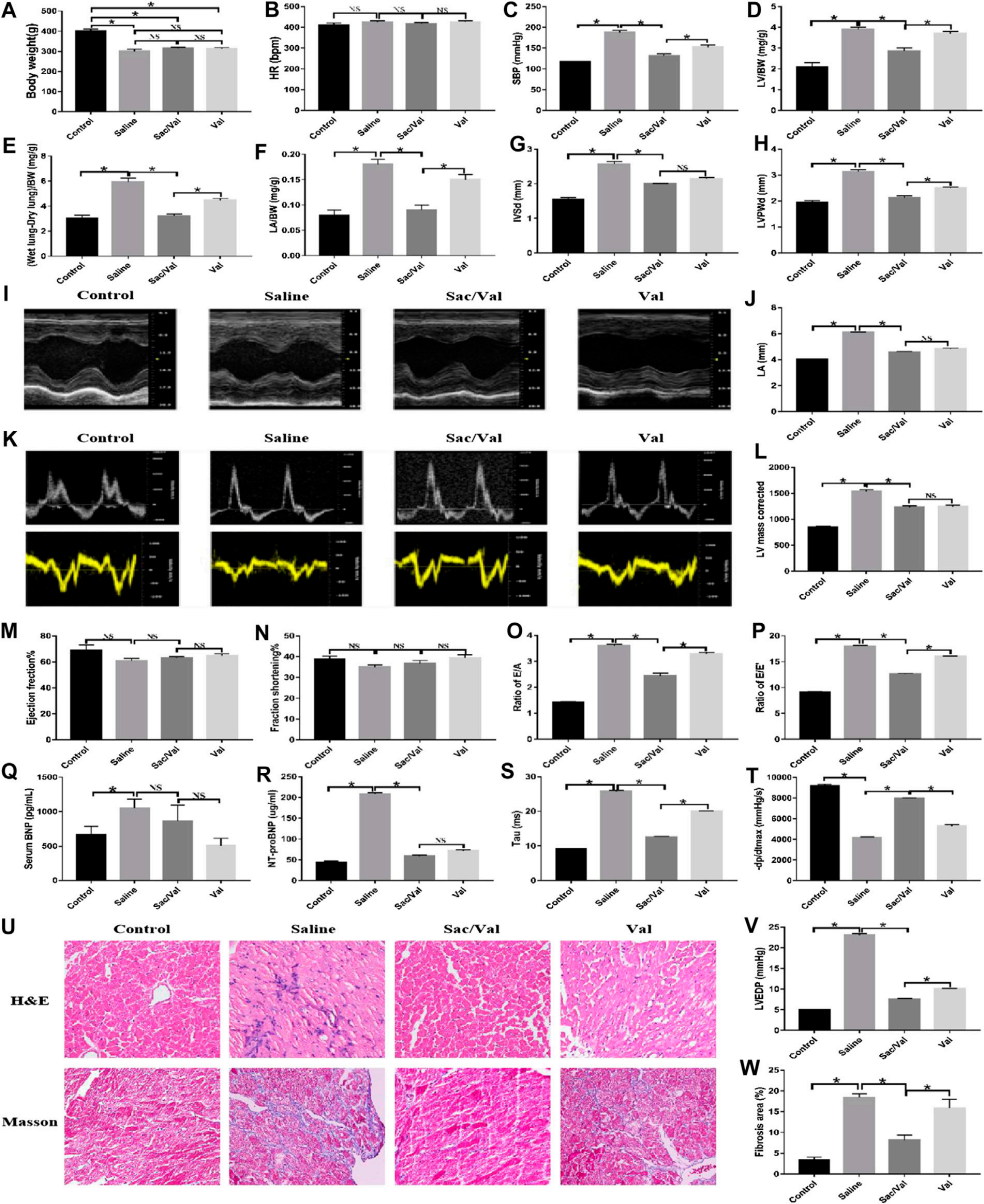
Treatment of Sac/Val in rats accompanied with HFpEF shows an obvious improvement of cardiac function of the rats in 23 weeks. **(I, K)** Measurements of cardiac function of rats were conducted by M-mode ultrasound and color Doppler ultrasound of the echocardiography (n = 10 in each group). **(Q, R)** ELISA analysis of BNP and NT-ProBNP levels in serum of rats. **(A–H, J, L, M–P, S, T, V)** The parameters (body weight, HR, SBP, LV/BW, (Wet lung-Dry lung)/BW, LA/BW, IVSd, LVPWd, LA, LV mass corrected, EF%, FS%, E/A, E/E′, Tau, −dp/dtmax, and LVEDP) were collected to evaluate the treatment of Sac/Val in rats of HFpEF. **(U)** H&E and Masson’s trichrome staining of heart tissues. **(W)** Assessment of cardiac fibrosis by Masson’s trichrome staining. Quantification of fibrotic areas: *p < 0.05 means statistic significance; NS means no statistic significance. All these data represent the mean ± SD of at least three times of the experiment. Tau, left ventricular relaxation time constant; −dp/dtmax, maximum left ventricular pressure decreasing rate; LVEDP, left ventricular end-diastolic pressure.

After 4 weeks of treatment in respective groups, we found a significant reduction in BW in the Saline, Sac/Val, and Val groups (*p* < 0.05), compared with the Control group; however, there was an insignificant difference (*p* > 0.05) in BW among the three groups (Saline, Sac/Val, and Val groups) (Figure 2A). Additionally, there was no significant difference (*p* > 0.05) in the HR between all four groups (Figure 2B). Nevertheless, the rats in the Saline group exhibited elevated levels of SBP, LV/BW, (Wet lung-Dry lung)/BW, and LA/BW (*p* < 0.05) compared with the Control group, and treatment with Sac/Val and Val substantially decreased these indices (*p* < 0.05) with Sac/Val being more efficient than Val (Figures 2C–F). These observations indicated that treatment with Sac/Val could significantly attenuate the symptoms of high-salt diet-induced HFpEF in rats and its protective effects were better than Val alone.

The results of echocardiography reiterated the better protective effects of Sac/Val over Val alone for the treatment of high-salt diet-induced HFpEF. Compared with the Control group, the Saline group exhibited cardiac dysfunction related to cardiac hypertrophy or diastolic dysfunction, based on an increase in several indices, such as IVSd, LVPWd, LA, corrected LV mass, E/A and E/E′. There was a significant improvement in these parameters after treatment with Sac/Val and Val (*p* < 0.05), with Sac/Val showing a stronger therapeutic effect than Val, although there was only a minor change in EF and FS between these groups (Figures 2G–P). Compared with the Control group, there was a significant increase in the serum levels of BNP and NT-ProBNP (*p* < 0.05) in the Saline group. By treatment with Sac/Val, the serum level of BNP maintained a close level to the Saline group while NT-ProBNP was reduced significantly (*p* < 0.05) (Figures 2Q,R). The rats in the Saline group had a minimal left ventricular diastolic function, as measured by the carotid artery surgery, based on indices, such as LVEDP, Tau, and −dp/dt_max_; however, treatment with Sac/Val reversed this dysfunction (Figures 2S,T,V). Similarly, H&E staining of the tissue specimens from the Saline group showed apparent edema and disordered arrangement of myofilaments, and Masson’s trichrome staining exhibited increased fibrosis compared with the Control group. These pathological changes were markedly improved (*p* < 0.05) by treatment with Sac/Val (Figures 2U,W). These observations suggested that Sac/Val could significantly alleviate the symptoms associated with high-salt diet-induced HFpEF and Sac/Val could serve as a novel therapeutic agent for the treatment of HFpEF.

### Treatment With Sac/Val Alleviated Vascular Injury Related to High-Salt Diet-Induced HFpEF

**FIGURE 3:**
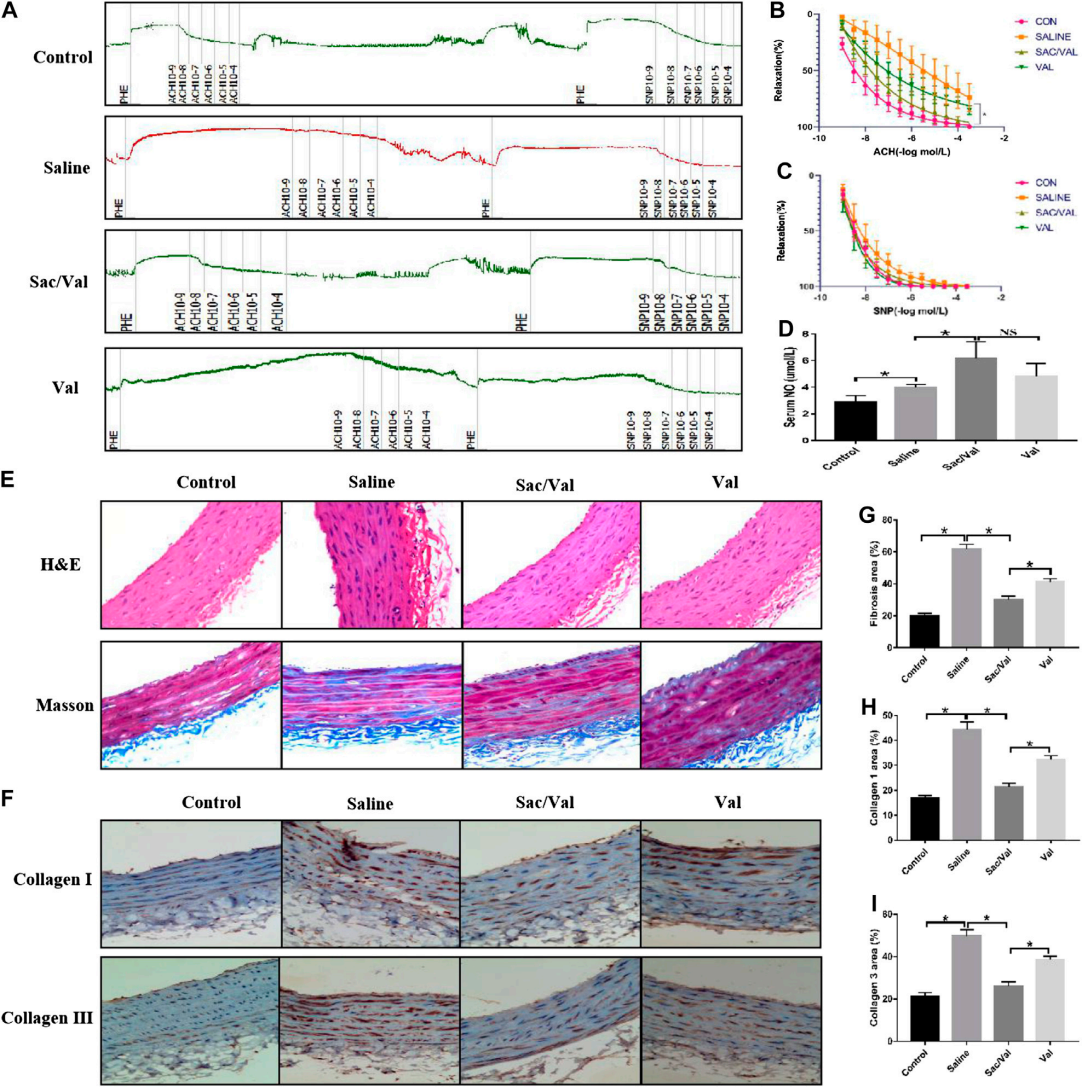
Treatment of Sac/Val in rats accompanied with HFpEF significantly alleviated the vascular injury of HFpEF induced by high-salt diet. **(A)** The primary procedures of evaluating the vascular endothelium diastolic function (n = 10 in each group). **(B, C)** Acetylcholine (Ach) is used to measure endothelium-dependent vasodilation and sodium nitroprusside (SNP) to measure endothelium-independent vasodilation. **(D)** The levels of NO in serum of rats were detected to evaluate the vascular injury. **(E)** H&E and Masson’s trichrome staining were used to assess the pathological changes of blood vessel. **(G)** Assessment of vascular fibrosis by Masson’s trichrome staining; quantification of fibrotic areas. **(F, H, I**) Immunohistochemical staining of Collagen 1 and Collagen 3 in blood vessel. Quantification of Collagen 1 and Collagen 3 areas: *p < 0.05 means statistic significance; NS means no statistic significance. All these data represent the mean ± SD of at least three times of the experiment.

Previous studies have shown that vascular injury is a pathological change associated with the development of HFpEF (Franssen et al., 2016). Thus, healing of the vascular injury would imply an improvement in the symptoms of high-salt diet-induced HFpEF. The carotid artery isolated from rats was used to evaluate the vascular endothelium diastolic function. Figure 3A summarizes the relevant primary procedures. The measurement of Ach-induced endothelium-dependent vasodilation function revealed that the high-salt diet in the Saline group significantly impaired (*p* < 0.05) the vascular endothelium diastolic function compared with the Control group, and this effect was markedly inhibited (*p* < 0.05) by treatment with Sac/Val and Val. As expected, Sac/Val showed a stronger inhibition than Val (Figure 3B). However, there was no significant difference among the four groups related to SNP-induced endothelium-independent vasodilation function (*p* > 0.05) (Figure 3C). We observed a significant increase in the serum levels of NO in rats with HFpEF treated with Sac/Val, which revealed a better protective effect than Val (Figure 3D). H&E and Masson’s trichrome staining were performed to assess the histological changes in the vascular endothelium of these rats. The result of H&E staining showed that the vascular endothelium was seriously damaged in the Saline group than in the Control group, and this effect was significantly reversed in the Sac/Val group (Figure 3E). Masson’s trichrome staining revealed that the treatment with Sac/Val significantly decreased (*p* < 0.05) the level of fibrosis in vascular endothelium induced by the high-salt diet and was better than treatment with Val (Figures 3E,G). Similarly, the IHC analysis of type I collagen and type III collagen further confirmed that treatment with Sac/Val significantly inhibited (*p* < 0.05) the high-salt diet-induced fibrosis in the vascular endothelium of the rats (Figures 3F,H,I). Thus, Sac/Val protected the tissues against vascular injury associated with high-salt diet-induced HFpEF via inhibiting the fibrotic reaction and was potent than Val.

### Treatment With Sac/Val Inhibited High-Salt Diet-Induced Cardiac Fibrosis

**FIGURE 4:**
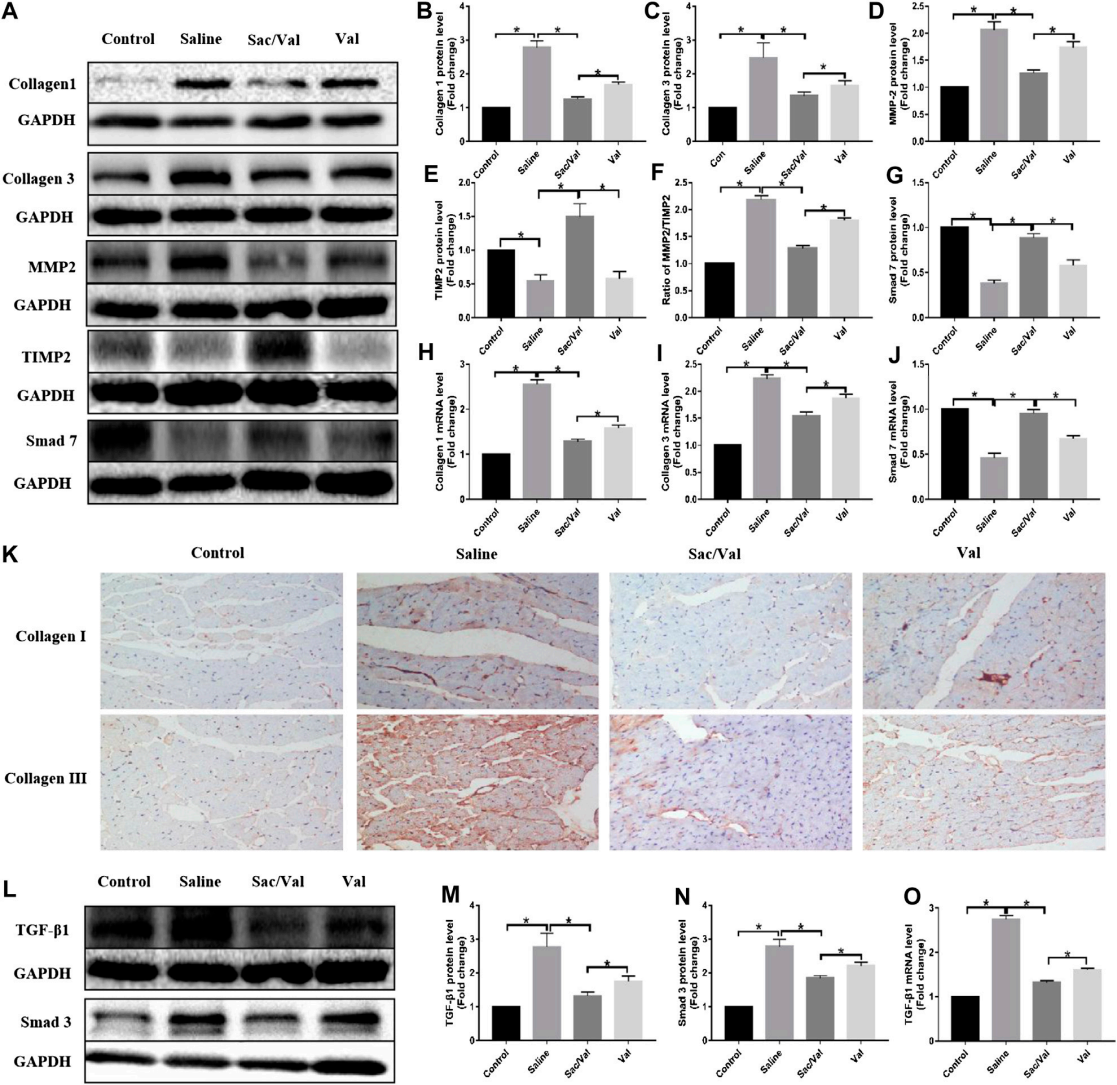
Treatment of Sac/Val can inhibit cardiac fibrosis via TGF-β1/Smad3 signaling pathway potentially. **(A, L)** Western blot analysis of protein expression levels in rats heart tissues (n = 10 in each group). The proteins include Collagen 1, Collagen 3, MMP2, TIMP2, Smad 7, TGF-β1, and Smad3. **(B–E, G)** Statistic analysis of protein expression levels of Collagen 1, Collagen 3, MMP2, TIMP2, and Smad 7. **(F)** The ratio of MMP2/TIMP2 was calculated. **(H–J)** qRT-PCR analysis of Collagen 1, Collagen 3, and Smad 7 mRNA expression levels in heart tissues. **(K)** Immunohistochemical staining of Collagen 1 and Collagen 3 in heart tissues. **(M, N)** Statistic analysis of protein expression levels of TGF-β1 and Smad3. **(O)** qRT-PCR analysis of TGF-β1 mRNA expression level in heart tissues. *p < 0.05 means statistic significance; NS means no statistic significance. All these data represent the mean ± SD of at least three times of the experiment.

Next, we evaluated the expression of fibrotic genes in all four groups to elucidate the effects of Sac/Val on high-salt diet-induced cardiac fibrosis. The results showed a significant increase in the protein and mRNA expression of both type I collagen and type III collagen (*p* < 0.05) in heart tissue specimens of rats fed with high-salt feed compared with the Control group rats, while the effects were significantly inhibited (*p* < 0.05) by treatment with Sac/Val and Val, with the former being more potent than the latter (Figures 4A,B,C,H,I). Moreover, IHC analysis of type I collagen and type III collagen further confirmed that treatment with Sac/Val significantly inhibited high-salt diet-induced cardiac fibrosis in the heart tissue specimens (Figure 4K). We also studied the protein expression of MMP2 and TIMP2 and found a significant decrease in the ratio of MMP2/TIMP2 (*p* < 0.05) in the Sac/Val group than in the Saline group and the Val group (Figures 4A,D–F). Therefore, Sac/Val exerted an antifibrotic role that protected against high-salt diet-induced cardiac fibrosis.

The TGF-β/Smad signaling pathway plays an important role in cardiac fibrosis. Thus, Western blot and qRT-PCR were performed to evaluate the effects of treatment with Sac/Val on the expression of components of TGF-β/Smad. The high-salt diet resulted in upregulated expressions of TGF-β1 and Smad3, while these effects were inhibited by treatment with Sac/Val except for Smad7 (Figures 4A,G,J,L–O), suggesting that the TGF-β1/Smad3 signaling pathway was probably involved in the protective effects exhibited by Sac/Val on high-salt diet-induced cardiac fibrosis.

## Discussion

Here, we successfully constructed an animal model of HFpEF by feeding Dahl/SS rats with high-salt feed until 19 weeks (Doi et al., 2000). The results showed that treatment with Sac/Val exerted a protective effect on high-salt diet-induced HFpEF coupled with vascular injury, and this effect was more potent than Val, implicating that Sac/Val could serve as a novel therapeutic agent for the treatment of HFpEF.

Sac/Val is a combination therapeutic agent containing sacubitril and valsartan (1:1), which is the first ARNI. This combination was created to benefit from neprilysin inhibitor (NEPI) and angiotensin receptor blockers (ARB) by decreasing the risk of angioedema induced by angiotensin-converting enzyme inhibitors (ACEI) (Akbar et al., 2020). Several studies have reported the therapeutic efficiency of Sac/Val, also known as LCZ696, in the treatment of HFrEF (Giallauria et al., 2020). The global PARADIGM-HF randomized trial concluded that Sac/Val markedly reduced the rates of mortality and HF-related hospitalization in HFrEF by approximately 20% and the all-cause mortality rate by approximately 16% (McMurray et al., 2014). Treatment with Sac/Val showed clinical efficacy in patients with HFrEF having mild to moderate symptoms. However, Douglas et al. found that in the global PARADIGM-HF randomized trial, less than 1% of patients with advanced HF that were treated with Sac/Val experienced limited benefits. Thus, a novel study, called LIFE (LCZ696 In Hospitalized Advanced Heart Failure) trial, was designed to confirm the results of PARADIGM-HF (Mann et al., 2020). Moreover, Evgeniya et al. discovered that Sac/Val improved cardiac function and decreased cardiac fibrosis by downregulating the exosomal miR-181a in rats with HF caused by myocardial infarction (MI) (Vaskova et al., 2020). Due to these significant therapeutic effects of Sac/Val in patients with HFrEF, several important international medical institutions, such as European Medicines Agency (EMA), Food and Drug Administration (FDA), American Heart Association (AHA), and New York Heart Association (NYHA), have approved Sac/Val as a replacement of ACE inhibitors for treating HFrEF patients (Fala, 2015; Yancy et al., 2017). HFpEF and HFrEF are known to share some pathophysiological characteristics, such as cardiac fibrosis (Hahn et al., 2020). Thus, we conducted this study to investigate whether Sac/Val could relieve symptoms of HFpEF. These results showed that Sac/Val had a positive effect on high-salt diet-induced HFpEF coupled with vascular injury.

First, we performed several functional tests using echocardiography, a BP-2010E noninvasive rat tail sphygmomanometer, a multichannel biological recorder, and DMT620 to evaluate the functioning of the heart and vascular endothelium. Sac/Val significantly alleviated the dysfunction of the heart and vascular endothelium and was more potent than Val (Kusaka et al., 2015; Trivedi et al., 2018). In recent years, serum BNP concentration has been increasingly used as a marker in patients with heart failure, which displays a protective function in heart failure (Ozhan et al., 2007). Jonathan W et al. recently found that NT-ProBNP acted as a strong predictive factor of HF events in patients with HFpEF and Sac/Val could significantly reduce the levels of NT-ProBNP in patients with HFpEF (Cunningham et al., 2020). NO acts as an important marker of endothelial injury and exerts a protective function by regulating the tension in vascular endothelium (Verleden et al., 1999). Consistent with these findings, we discovered a significant decrease in the serum levels of NT-ProBNP in the animal model of HFpEF despite no statistically significant changes in the serum levels of BNP, while an increase in the serum levels of NO was observed after treatment with Sac/Val, based on the results of ELISA. Next, H&E staining, Masson’s trichrome staining, and IHC were used to analyze the pathological changes in the tissue specimens of the heart and vascular endothelium, both of which showed a better therapeutic effect after treatment with Sac/Val. Thus, these data indicated that Sac/Val served as a novel therapeutic agent for the treatment of HFpEF coupled with vascular injury. We further performed Western blot and qRT-PCR to analyze the molecular mechanism of Sac/Val and found that the mRNA and protein expressions of type I collagen and type Ⅲ collagen in the heart tissue specimens were significantly inhibited after treatment with Sac/Val, indicating that Sac/Val protected against HFpEF by inhibiting cardiac fibrosis. The TIMP2 protein is a specific inhibitor of MMP2, a pro-fibrotic protein, and a high ratio of MMP2/TIMP2 represents aggravated fibrosis (Ding et al., 2014). Our findings confirmed the protective role of Sac/Val, evidenced by the lower ratio of MMP2/TIMP2. The TGF-β/Smad signaling pathway is known to play an important role in the development of cardiac fibrosis (Hu et al., 2020). Therefore, we speculated that the TGF-β/Smad signaling pathway might be involved in the antifibrotic role of Sac/Val in high-salt diet-induced cardiac fibrosis. Our findings showed that Sac/Val markedly suppressed the activation of the TGF-β1/Smad3 signaling pathway during the progression of the high-salt diet-induced cardiac fibrosis. The expression of Smad7, which is a feedback inhibitor of TGF-β, was increased by treatment with Sac/Val (Cho et al., 2020), suggesting that this pathway was probably involved in the cardioprotective effects of Sac/Val.

However, this study had several limitations. First, the study only determined the effects of a high-salt diet and Sac/Val on the TGF-β/Smad signaling pathway at the molecular level but did not determine the agonists or antagonists involved in this signaling pathway to confirm the results. Thus, further research is required to identify the role of this signaling pathway in high-salt diet-induced HFpEF. Additionally, *in vitro* experiments are also required for the comprehensive verification of these results.

Thus, this study suggested that Sac/Val had a positive role in alleviating the symptoms of HFpEF coupled with vascular injury, which was more effective than Val. The protective abilities were probably derived from the inhibition of cardiac fibrosis by suppressing the TGF-β1/Smad3 signaling pathway. Thus, our findings implied that Sac/Val could serve as a more promising therapeutic agent for treating HFpEF.

## Data Availability Statement

The raw data supporting the conclusions of this article will be made available by the authors, without undue reservation, to any qualified researcher.

## Ethics Statement

The animal study was reviewed and approved by The Animal Care and Use Committee of the Second Affiliated Hospital of Nanchang University (China). Written informed consent was obtained from the owners for the participation of their animals in this study.

## Author Contributions

YD designed the present study. WeZ and YF conducted the experiments and analyzed the data. WeZ and YF interpreted the results and prepared the manuscript. YF, WeZ, and JL reviewed the paper. All other authors supported the study and agreed on the final manuscript.

## Funding

The present study was funded by the National Natural Science Foundation of China (81960088 and 81460071), and Innovation driven “5511” project platform and talent team project, Jiangxi, China (20165BCB18020).

## Conflict of Interest

The authors declare that the research was conducted in the absence of any commercial or financial relationships that could be construed as a potential conflict of interest.
