# Supplementary figures and images for: Sacubitril/Valsartan Reduces Fibrosis and Alleviates High-Salt Diet-Induced HFpEF in Rats
Source: Front Pharmacol. 2021 Jan 14;11:600953. doi: 10.3389/fphar.2020.600953 (PMC7841406; doi:10.3389/fphar.2020.600953)

Collagen 1

1：


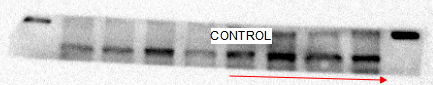

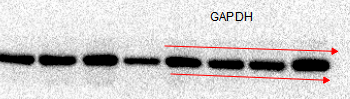


2：


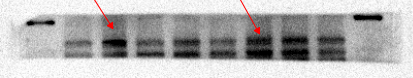


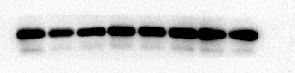


Collagen 3

1：


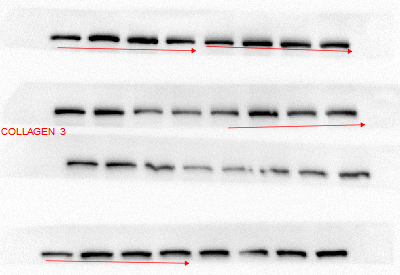

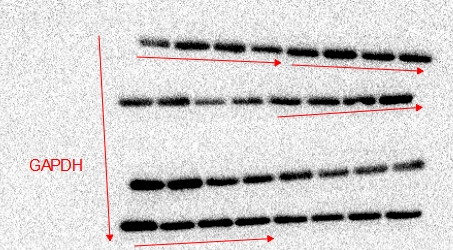


2：


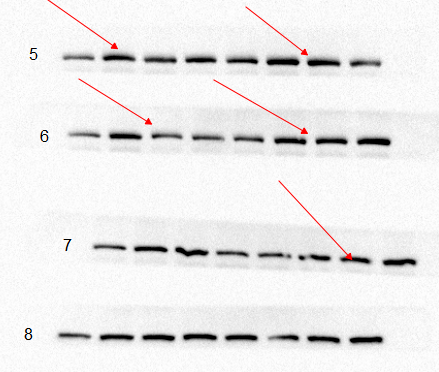

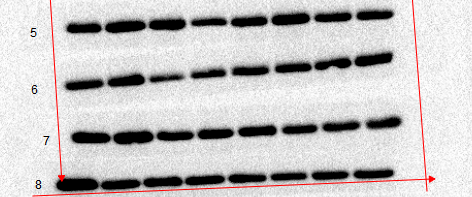


MMP2

1:


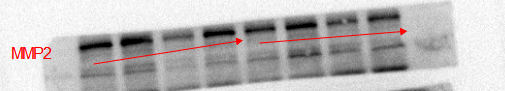

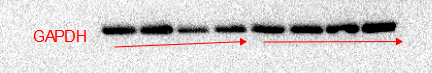


2:


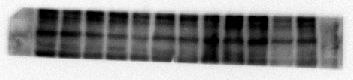

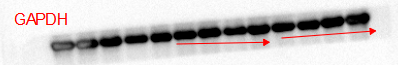


TIMP2

1:


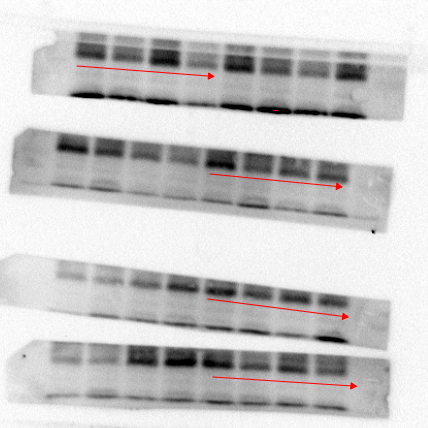


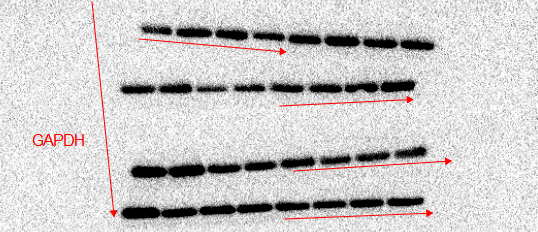


SMAD 7

1:


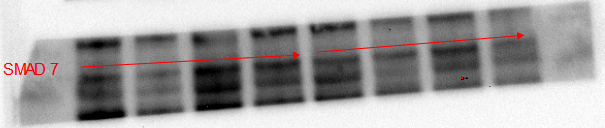


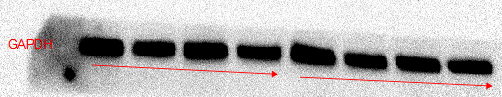


2:


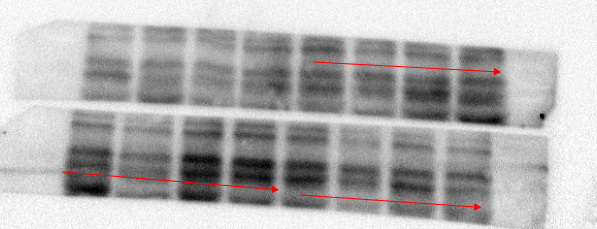


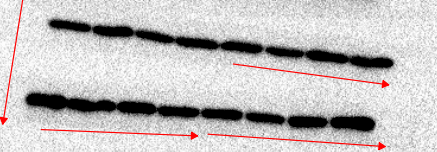


TGFB-1

1:


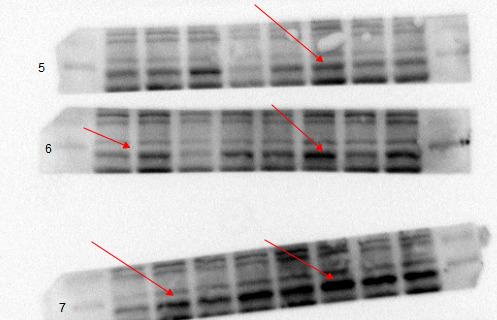

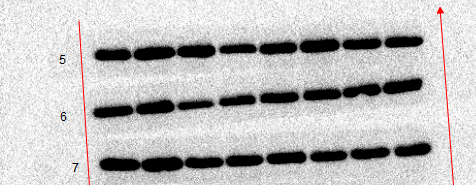


SMAD 3

1:


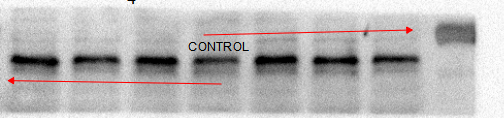

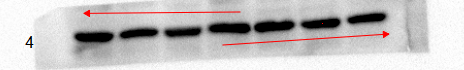


2:


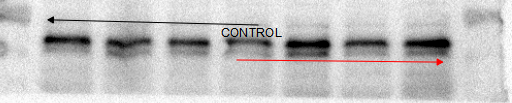

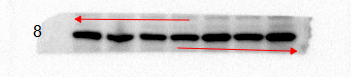

Supplement: Supplementary file 2 [file Table1.doc]
